# Polymorphisms Affecting the Response to Novel Antiepileptic Drugs

**DOI:** 10.3390/ijms24032535

**Published:** 2023-01-28

**Authors:** Valentina Urzì Brancati, Tiziana Pinto Vraca, Letteria Minutoli, Giovanni Pallio

**Affiliations:** Department of Clinical and Experimental Medicine, University of Messina, Via C. Valeria, 98125 Messina, Italy

**Keywords:** epilepsy, novel antiepileptic drugs, AEDs, pharmacogenetics, SNPs

## Abstract

Epilepsy is one of the most frequent chronic neurologic disorders that affects nearly 1% of the population worldwide, especially in developing countries. Currently, several antiepileptic drugs (AEDs) are available for its therapy, and although the prognosis is good for most patients, 20%–30% amongst them do not reach seizure freedom. Numerous factors may explain AED-resistance such as sex, age, ethnicity, type of seizure, early epilepsy onset, suboptimal dosing, poor drug compliance, alcohol abuse, and in particular, genetic factors. Specifically, the interindividual differences in drug response can be caused by single nucleotide polymorphisms (SNPs) in genes encoding for drug efflux transporters, for the brain targets of AEDs, and for enzymes involved in drug metabolism. In this review, we used the PubMed database to retrieve studies that assessed the influence of SNPs on the pharmacokinetic (PK), pharmacodynamic (PD), and efficacy of new antiepileptic drugs. Our results showed that polymorphisms in the ABCB1, ABCC2, UGT1A4, UGT2B7, UGT2B15, CYP2C9, and CYP2C19 genes have an influence on the PK and efficacy of AEDs, suggesting that a genetic pre-evaluation of epileptic patients could help clinicians in prescribing a personalized treatment to improve the efficacy and the safety of the therapy.

## 1. Introduction

Epilepsy is one of the most frequent chronic neurologic disorders, and it affects nearly 1% of the population worldwide, especially in developing countries [1]. It is characterized by recurrent epileptic seizures caused by super-synchronous discharges in the brain neurons [2]. Currently, around 25 antiepileptic drugs (AEDs) are available for epilepsy therapy [3], and they are classified as old or first generation and the new generation of antiepileptics. The older-generation AEDs have been used in clinical practice for more than four decades, and they include valproate, carbamazepine phenobarbital, ethosuximide, phenytoin, primidone, clonazepam, and clobazam [4] (Table 1), while the new generation AEDs have been approved for the therapy of epilepsy since 1989. In particular, they are divided into second generation and third generation, which have been launched in the market since 2004 [5] (Table 1). Older AEDs such as phenytoin, carbamazepine, valproic acid (sodium valproate), and phenobarbital are characterized by a narrow therapeutic range and a pronounced inter-individual variability in their pharmacokinetics. It is often claimed that the new generation of AEDs such as gabapentin and levetiracetam have a major advantage over the older AEDs in that they have more predictable pharmacokinetics [6].

Moreover, depending on their mechanism of action, the AEDs can be classified in four main categories: (i) voltage-gated ion channels modulators; (ii) neurotransmitter-release modulators; (iii) GABAergic transmission enhancers; and (iv) glutamatergic transmission inhibitors [4].

Amongst the AEDs that block ion channels, the most important class are the voltage-gated sodium channel blockers. These drugs (i.e., lamotrigine and lacosamide) act to prevent the opening of membrane depolarization-activated sodium channels, thus arresting the initiation of action potentials [7]. Regarding the neurotransmitter release modulators, the most widely used drugs are levetiracetam and brivaracetam, and both these compounds facilitate the action of synaptic vesicle protein 2A (SV2A) [8,9], a protein that reduces the fusion between the vesicle and presynaptic membrane by altering the cell calcium responsivity [10]. Moreover, barbiturates, benzodiazepines, vigabatrin, and tiagabine are the main actors in the modulation of GABAergic transmission. The first two drugs act directly on the GABAA channel, which, when open, leads to a membrane hyperpolarization by allowing a chloride current within the cell. On the other hand, tiagabine and vigabatrin act by increasing the quantity of GABA available in the synaptic cleft. Finally, the only molecule that specifically acts on glutamatergic receptors is perampanel [11], a specific non-competitive AMPA receptor antagonist, even if an antagonistic action on the metabotropic mGluR5 receptor has also been suggested as an associated mechanism of action of rufinamide.

Although these drugs ameliorate the prognosis for most patients affected by epilepsy, 20%–30% of subjects do not reach seizure freedom, even when treated with multiple AEDs [12]. Several factors may be addressed to partly explain AED-resistance such as early epilepsy onset, type of seizure, suboptimal dosing, poor drug compliance, alcohol abuse, and a high frequency of seizures in the diagnostic assessment period [13]. In addition, it has been suggested that genetic factors may contribute to this variability in clinical outcomes [14]. In fact, single nucleotide polymorphisms (SNPs), which are variations of a single DNA base, may affect the AEDs’ efficacy, tolerability, safety, and duration of action [15]. Specifically, the interindividual variability in drug response can be affected by SNPs in genes encoding for drug efflux transporters (ABCB1, ABCC2) localized in the gastrointestinal tract and blood–brain barrier (BBB), for brain targets of AEDs (voltage-dependent Na+ channels, synapse vesicle protein SV2A), and for enzymes involved in drug metabolism (CYP2C19, UGT1A4).

Therefore, the aim of the present review was to evaluate the effects of SNPs in genes involved in the pharmacokinetic (PK) and pharmacodynamic (PD) of AEDs and assess if these polymorphisms could affect the efficacy, blood levels, and clinical outcomes of AEDs in epileptic patients. In particular, we chose to focus our attention on the second and third generation of antiepileptic drugs in light of their rapid increase in clinical practice.

## 2. Polymorphisms Affecting AEDs Transporters

SNPs in genes encoding for drug efflux transporters may affect the absorption and distribution of AEDs, and the most studied polymorphisms are those concerning the genes encoding for the transporters ABCB1 and ABCC2, also known as P-glycoprotein (P-gp), which transport several AEDs [16]. One of the most common variants in the ABCB1 gene is the C3435T polymorphism in exon 26 of the MDR1(Multi-Drug Resistance 1) gene, which was correlated with drug resistance in epilepsy among Caucasians. In fact, it has been observed that individuals with 3435 TT carriers have reduced P-gp activity and higher plasma drug concentrations after oral administration [17,18]. P-gp is present in duodenal cells, and this explains why ABCB1 SNPs can influence the absorption and plasma concentrations of AEDs [19]. Moreover, P-gp is also present in the membrane of cerebral capillary endothelial cells, in order to protect the brain from intoxication caused by lipophilic xenobiotics. Therefore, an increased expression of this gene causes higher amounts of P-gp in the endothelial cells and in astrocytes, leading to a decreased drug parenchymal concentration, regardless of the fact that they reach blood therapeutic levels [20]. In this context, Shen et al. found that carriers of the ABCB1 3435C > T CC genotype had a higher oxcarbazepine concentration than TT. They also observed more oxcarbazepine-responsive patients amongst the CC genotype than CT and TT. However, when ABCC2 SNPs were taken into account, they did not find an effect on the oxcarbazepine plasma concentrations and therapeutic efficacy [21]. On the other hand, an influence of ABCC2 1249 G > A on the oxcarbazepine dose was observed in a study conducted by Ma et al., which showed that ABCC2 1249 G > A carriers needed a significantly higher oxcarbazepine dose than non-carriers [22].

**Figure 1:**
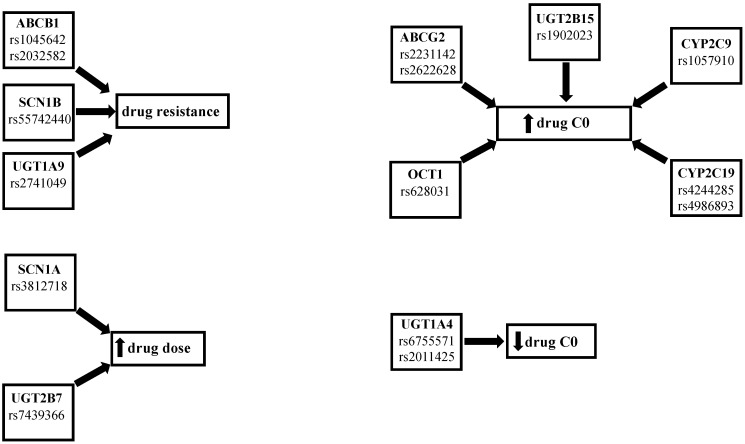
Summary of the effects of the SNPs.

Moreover, Yao et al. found that child carriers of the ABCB1 C3435 > T CC genotype had a higher concentration–dose ratio (CDR) of oxcarbazepine when compared with CT carriers [23]. Two studies by Zhao and colleagues assessed lacosamide PK and drug-resistance in a population of Asiatic pediatric patients. The first one showed that patients with ABCC2 1249G > A GA and AA genotypes and with ABCC2 -24C > T CT and TT genotypes had lower lacosamide CDR than the ABCC2 1249G > A GG and ABCC2 -24C > T CC genotypes [24]. In the second study, they found that the drug-resistant group had a higher frequency of the ABCB1 G2677T/A GT genotype, while a higher CDR value was found in the GG genotype and in the ABCB1 C3435T CC genotype when compared to the G2677T/A AT and ABCB1 C3435T CT genotypes. Moreover, they observed higher lacosamide plasma levels in ABCB1 C3435T CC carriers rather than the CT and TT carriers [25]. Furthermore, Zhou et al. reported that rs3114020 TT carriers had lower lamotrigine CDR values than C allele carriers and patients carrying the rs2231142 CA or AA genotype showed higher lamotrigine concentrations compared with the CC carriers [26]. Shen et al. studied the association between SNPs in ABCG2 or ABCC2 genes and lamotrigine pharmacokinetics and pharmaco-resistance. They also investigated the influence on these parameters of SNPs in another subfamily of influx transporters, the organic cation transporters (OCTs), and of the hepatocyte nuclear factor 4 alpha (HNF4a), a liver-enriched regulator of liver development that binds several gene promoters in human and mouse liver including CYPs, UGTs, and transporters [27,28]. They reported higher lamotrigine levels in the patients that were carriers of the rs628031 AA and AG genotype and in the rs2231142 AA carriers when compared with the OCT GG genotype and ABCG2 CC and CA genotype, respectively. On the other hand, they found no significant influence of the other studied SNPs (OCT1 rs2282143, ABCG2 rs2231137, ABCC2 rs2273697, and HNF4 rs2071197, rs3212183) on lamotrigine concentrations and therapeutic response [29]. Lovric et al. studied the influence on lamotrigine levels of ABCB1 C1236T, G2677T/A, and C3435T SNPs. They reported that 1236CT and TT genotype carriers had lower lamotrigine levels and dose-corrected concentrations than CC carriers. They also found no significant influence on the lamotrigine parameters of ABCB1 G2677T/A and the C3435T genotypes, although the concentrations in the monotherapy group were higher in the 2677GG and C3435T CC genotypes [30]. Two studies analyzed the correlation between SNPs in transporters and the pharmaco-resistance of AEDs. In the first, Stasiołek et al. found that ABCB1 C3435T CC carriers had a higher incidence of drug resistant epilepsy in a population of Polish children [31]. In the second, Ufer et al. observed, in a Caucasian population, a higher frequency of the ABCC2 1249G > A variant in the responder’s patients. Instead, they reported that ABCC2 -24C > T and 3972C > T did not influence therapy response [32]. On the other hand, some studies have reported a lack of association between polymorphisms in transporter genes and the effects of AEDs. For example, Wang et al. did not find a significant correlation between SNPs in the ABCB1 genes and oxcarbazepine PK [33]. Lin et al. studied the ABCB1 3435C > T and ABCB2 1249G > A genotypes and found that they had no influence on oxcarbazepine PK in a pediatric population [34]. Petrenaite et al. found no association between the ABCB1 1236 C > T and ABC1B1 3435 C > T SNPs and lamotrigine [35]. Finally, Zhao et al. did not find a correlation between the presence of the ABCB1 C1236T genotype and the levetiracetam levels [36]. In summary, several studies found a correlation between the presence of SNPs in the ABCB1, ABCC2, and ABCG2 genes and PK, PD, or efficacy of AEDs, which suggests that a pharmacogenetic pre-evaluation could be useful for prescribing the most appropriate dose-drug, according to the patient’s genetic profile (Figure 1 and Table 2).

## 3. Polymorphisms Affecting AED Brain-Targets

Regarding the brain targets of the AEDs, the most interesting data up until now have been reported on voltage-dependent Na+ channels (SCNs), which are responsible for the generation and propagation of action potential in neurons, and for this reason, many AEDs act by reducing the high-frequency firing of the voltage-dependent SCN that occurs during seizures [37]. SCN are formed by one alpha subunit and two beta subunits. The alpha subunits are encoded by the SCN (1–10)A genes and beta subunits by the SCN(1–4)B genes [38]. Exon 5 of the SCN1A gene encodes for the I-S4 voltage sensor domain, one of the sodium channels expressed in brain cells. Genetic variations in the α-subunit may affect the electro-physiological properties of sodium channels in drug-resistant epilepsy patients. For example, it has been suggested that the SCN1A-A3184G (p.Tr1067Ala) polymorphism may be involved in the gating of sodium channels, therefore making them insensitive to sodium-channel blockers [39].

In this regard, Lin et al. studied the influence of SCN1A, SCN1B, SCN2A, and SCN9A SNPs on the response to several sodium blocker AEDs in 214 Taiwanese patients. They observed that non-responder patients had a significantly higher frequency of allele C of rs55742440 in SCN1B. Moreover, they found no significant association between SNPs and AED response in the predominant sodium channel blocking AED group, which included oxcarbazepine, lamotrigine, and lacosamide [40]. Moreover, Angelopoulou and colleagues reported a similar distribution of SCN1A IVS5-91 rs3812718 G > A genotypes between drug-responsive and drug-resistant patients treated with sodium channel blocker AEDs. They also found that within the monotherapy-responsive group, G/G carriers needed lower doses than A/A or A/G carriers [41]. Furthermore, Gazhala et al. studied the SCN1A-A3184G SNP in 326 children with non-lesional epilepsy, 163 AEDs-resistant, 163 AEDs responders, and 163 healthy controls, and they did not find a significant influence of the studied SNPs between the AED responders and those resistant [42]. Pejanovic-Skobic found no significant difference of SCN2A 56G > A regarding G allele frequency between the responders or no responders to lamotrigine monotherapy, and between epileptic patients and healthy control subjects [43]. Ma et al. found that SCN1A IVS5-91G > A variant allele carriers needed significantly higher oxcarbazepine maintenance doses than non-carriers [22]. Studying the same polymorphism, other studies did not find a significant influence on lamotrigine and oxcarbazepine response or maintenance dosage. Thus, Kumari and colleagues did not observe an effect of the SCN1A IVS5-91G > A genotype on drug response in patients that showed drug resistance to oxcarbazepine [44]. This SNP was also investigated by Markovic et al., who did not find a significant influence of SCN1A IVS5-91 G > A SNPs on lamotrigine efficacy. However, they suggest that these SNPs may affect the average maintenance dose because in the responder group, the AA genotype needed higher doses than the GA and GG carriers, even if the difference was not statistically significant [45]. Furthermore, Manna et al. investigated the relationship between the c.603-91G > A polymorphism and response to oxcarbazepine, and they did not find a significant difference in the frequency of the presence of the 603-91G > A genotype between the drug-resistant and drug-responsive patients, and between this polymorphism and the response to oxcarbazepine. Moreover, the same authors found no influence of the 603-91G > A genotype on the drug dose in both the whole group of patients or within the drug-resistant compared to the drug-responsive patients [46].

In addition to SCNs, another important AED target is the synaptic vesicle protein SV2A, the prevalent of three isoforms (SV2A, SV2B, and SV2C) in the brain, and site of action of levetiracetam and brivaracetam. It has been reported that homozygous SV2A knockout mice are normal at birth but fail to grow, experience severe seizures, and die prematurely. In heterozygous knockout mice, the same study reported a likelihood to have seizure 10-fold higher than wild-type animals [47]. However, Lynch et al. found no association between the SV2A, SV2B, or SV2C polymorphisms and levetiracetam levels [48]. In summary, some studies found that SCN1A IVS5-91G > A GG carriers needed lower AED dose and a study found a higher presence of the SCN1B rs55742440 genotype in no-responders to sodium channel blockers (Figure 1 and Table 3); however, considering that few pharmacogenetic studies in epileptic patients treated with SCNs are present in the literature, the role of these SNPs on the PK, PD, and efficacy of AEDs should be further investigated.

## 4. Polymorphisms Affecting AEDs Metabolizing Enzymes

Important SNPs in genes for enzymes responsible for drug metabolism are those concerning the UDP-glucuronosyltransferase (UGT) and CYP P450 isoenzymes. In fact, UGT isoenzymes metabolize oxcarbazepine in its main active metabolite 10-monohydroxy derivative (MHD) [49] and lamotrigine in lamotrigine-2-N-glucuronide (2-N-GLUC) [50], whilst the most important isoenzymes for the metabolization of AEDs are CYP3A4 and CYP2C19 [51]. In particular, UGT1A4*3 (142T > G, L48V) and UGT1A4*2 (70C > A, P24T) the most common SNPs involved in AED metabolism, caused a decreased glucuronidation, whereas the presence of one or two defective alleles CYP2C9*2, CYP2C9*3, CYP2C19*2, CYP2C19*3 in the CYP2C9 and CYP2C19 genes led to an increase in the drug concentrations [52,53]. In this regard, Lu et al. observed that UGT1A9 I399 C > T carriers in 124 Chinese patients treated with oxcarbazepine had significantly lower MHD plasma levels and worse seizure control compared to non-carriers [54]. Shen et al. found that UGT2B7 802T > C CC genotype carriers had a higher oxcarbazepine concentration than TT [21]. Opposite results were reported by Lin et al., who did not observe any influence on oxcarbazepine PK when the presence of UGT2B7 802T > C and UGT1A9 I399C > T SNPs was investigated [34]. One of these SNPs was also studied by Ma et al., who found that carriers of UGT2B7 802T > C variant alleles required significantly higher oxcarbazepine maintenance doses than noncarriers [22]. Moreover, Petrenaite et al. evaluated the impact of SNPs UGT1A4*2 70C > A, UGT1A4*3 142T > G, UGT2B7*2 802C > T, UGT2B15*2 253G > Ton lamotrigine PK. They observed lower lamotrigine ratios (LTG plasma concentration/LTG dose/weight) in wild-type UGT1A4*2 C, UGT2B7*2 TT, and UGT2B15*2 TT carriers compared with heterozygous C-carriers, CC, and GG carriers, respectively [35]. Another two studies observed a higher plasma lamotrigine concentration and better therapeutic efficacy in patients with the UGT1A4 142T > G TT polymorphism [55,56]. Moreover, Wang et al. found that carriers of the UGT1A4 -219C > T/-163G > A variant had a significantly higher adjusted lamotrigine levels compared to wild-type carriers. They also found no associations between the UGT1A4 142T > G SNPs and lamotrigine levels [52]. Concerning CYP P450 SNPs, Ahn et al. studied the effects of CYP2C19*2, CYPC19*3, and CYP2C9*3 genotypes on lacosamide PK. Based on CYPC19 genotypes, they stratified the patients in EMs (*1/*1), IMs (*1/*2 or *1/*3) as poor metabolizers (PMs) (*2/*2, *2/*3, *3/*3). They found significantly higher lacosamide concentrations and CDR in PMs, and the PM group presented the lowest proportion of lacosamide-resistant patients [57]. In a similar study, Okada et al. divided 99 Japanese patients in zonisamide extensive (EMs) and poor metabolizers (PMs) based on their CYP2C19 genotype. They observed lower zonisamide clearance in extensive metabolizers. They also found no influence of CYP3A5 SNPs on zonisamide clearance [58]. No influence of CYP3A4 and CYP3A5 SNPs on perampanel and oxcarbazebine was found by two other studies conducted in an Asian population [59]. Wang et al. did not find a significant correlation between the CYP3A4 and CYP3A5 genes and oxcarbazepine PK [33]. In summary, some studies demonstrated a correlation between the presence of SNPs in the UGT1A4, UGT1A9, UGT2B7, CYP2C9, 2CYP2C19 genes and the PK, PD, and efficacy of AEDs (Figure 1 and Table 4).

## 5. Discussion and Conclusions

Epilepsy is one of the most diffuse neurological diseases; it can affect people within a wide age range, reaching its incidence peaks in the first few years of life and in the elderly [36]. Nowadays, thanks to an ample selection of available AEDs, most of the patients are able to achieve seizure freedom, but nevertheless, this result is not possible for almost 30% of the subjects, even if under polytherapy treatment. This pharmacological variability between and within patients can be due to various causes including sex, age, ethnicity, type of seizure, and genetic factors [12,13].

In this paper, we reviewed studies that analyzed the influence of genetic polymorphisms on the PK, PD, and efficacy of the new antiepileptic drugs. Our results confirmed an important role played by SNPs in the ABCB1 and ABCC2 genes. In fact, ABCB1 3435C > T CC has been observed to cause higher lacosamide and oxcarbazepine MHD levels, and a study observed a higher frequency of this polymorphism in pharmaco-resistant patients. Moreover, ABCB1 G2677T/A caused higher levetiracetam levels and lacosamide resistance. Regarding ABCC2 SNPs, we observed lower lacosamide levels in the ABCC2 -24C > T CT and TT carriers. When we assessed the role of SNPs in genes encoding for the most important targets of new antiepileptic drugs, all but one of our results did not find a significant correlation between the SNPs in SCNs and SVA2 and the studied parameters. Finally, an influence of genetic polymorphisms in metabolizing enzymes has been observed. In fact, the UGT1A9 I399 C > T carriers had significantly lower MHD plasma levels and worse seizure control, the UGT2B7 802T > C CC genotype had a higher oxcarbazepine concentration, and the UGT2B7 802T > C variant alleles needed higher OXC maintenance doses than non-carriers. Our results also showed lower lamotrigine ratios in the wild-type UGT1A4*2 C, UGT2B7*2 TT, UGT2B15*2 TT, and UGT1A4 -219C > T/-163G > A carriers. In this regard, it is important to underline that several studies found a significant influence of UGT SNPs on the lamotrigine levels only after including other variables in their statistical analysis, and from the data observed in these studies, the most important factor influencing lamotrigine levels is the co-administration of enzyme inhibitors such as valproic acid [60,61,62,63,64]. Its use, in fact, can prolong lamotrigine half-life even by two-fold [65], while enzyme inducers like phenytoin can drastically shorten this parameter [66]. Regarding CYP P450 SNPs, we overall observed an influence of CYP2C9 and CYPSC19 but not of SNPs in the CYP3A4 and CYP3A5 genes on the PK of AEDs.

In light of all of these SNPs that could affect the AED plasma levels and efficacy, genotyping is a fascinating option when choosing therapy for epileptic patients. In fact, recent developments in understanding the genetic and neurobiological basis of epilepsy are prospecting a new era for the treatment of this disease, where testing for gene variations might help to improve the efficacy and safety of epilepsy therapies, allowing for specialists to identify the best drug and dose-adjustment for each patient, reducing the frequency of therapeutic drug monitoring [67].

In conclusion, our work showed that SNPs in enzymes and transporters influence the pharmacokinetics of AEDs, and therefore, the use of population pharmacokinetic modelling incorporating the genotypes of drug-metabolizing enzymes and transporters can be one of the most useful tools to facilitate the determination of individualized dosing regimens in AED therapy, and genetic and non-genetic factors affecting enzyme activity can be used reliably under such models by clinicians in selecting the best AED and dose-adjustment for their patients. Nonetheless, further pharmacogenetic studies in large populations and including various ethnic groups should be conducted to consolidate the importance of genotyping as a standard practice in epileptic patients.

## Figures and Tables

**Table 1:** Classification of AEDs.

AED	Generation	Mechanism of Action
Phenobarbital	First	Positive modulation of GABA_A_ receptors increases the opening time of chloride channels with the migration of ions into neuronal cells and hyperpolarization of the cell membrane.
Phenytoin	First	Prolongs fast-inactivated VG sodium channel state decreasing the sodium influx across the membrane, and reducing the firing of action potentials and the neuronal overexcitation.
Primidone	First	GABA receptor agonist that increases the opening time of chloride channels and hyperpolarization of the cell membrane.
Ethosuximide	First	Blocks T-type calcium currents (low-voltage activated).
Valproate	First	Blocks T-type calcium or sodium channel and inhibits histone deacetylase.
Carbamazepine	First	Enhancement of sodium channel inactivation by reducing the high-frequency repetitive firing of the action potentials.
Clonazepam	First	High-potency GABAA receptor agonist, decreases 5-HT utilization in the brain, and blocks the egress of 5-HIAA from the brain.
Clobazam	First	Increases the opening time of chloride channels, with the migration of ions into neuronal cells and the hyperpolarization of the cell membrane.
Vigabatrin	Second	Irreversible inhibitor of the GABA-degrading enzyme, GABA transaminase.
Oxcarbazepine	Second	Binds to sodium channels and inhibits high-frequency repetitive neuronal firing inhibition as well as the release of glutamate.
Lamotrigine	Second	Selectively binds and inhibits voltage-gated sodium channels, stabilizing presynaptic neuronal membranes inhibiting presynaptic glutamate and aspartate release
Gabapentin	Second	Binds to the α_2_δ subunit of VG calcium channels, thus decreasing the density of pre-synaptic voltage-gated calcium channels and subsequent release of excitatory neurotransmitters.
Felbamate	Second	Antagonism of the NMDA receptor reducing glutamatergic transmission; inhibition of GABA-receptor binding and of voltage-gated sodium channels as well as calcium channels.
Topiramate	Second	Antagonism of AMPA/ GluR5 kainate receptors and increases GABA activity.
Tiagabine	Second	GABA reuptake inhibition, it prolongs inhibitory postsynaptic potentials.
Levetiracetam	Second	Modulation of synaptic neurotransmitter release through binding to the synaptic vesicle protein SV2A.
Zonisamide	Second	Altering the fast inactivation threshold of voltage-dependent sodium channels, it reduces sustained high-frequency repetitive firing of action potentials; it also inhibits low-threshold T-type calcium channels in neurons.
Pregabalin	Third	Binds to the α_2_δ subunit of VG calcium channels, reducing the synaptic release of several neurotransmitters.
Fosphenytoin	Third	Modulation of voltage-gated sodium channels by prolonging the inactivation state of these channels.
Lacosamide	Third	Selectively enhances the slow inactivation of voltage-gated sodium channels and possibly interacts with ollapsing response mediator protein-2.
Rufinamide	Third	Modulation of activity in sodium channels, particularly prolongation of the inactive state.
Eslicarbazepine	Third	Inhibiting voltage-gated sodium channels (VGSC), especially in rapidly firing neurons.
Retigabine	Third	Positive allosteric modulator of the neuronal potassium channels KNCQ (Kv2 to 5).
Perampanel	Third	Non-competitive selective antagonist at the postsynaptic ionotropic alpha-amino-3-hydroxy-5-methyl-4-isoxazolepropionic acid (AMPA) glutamate receptor.
Brivaracetam	Third	Binds to SV2A, modulating synaptic GABA release.
Cannabidiol	Third	Enhances GABA activity through allosteric modulation of the GABA_A_ receptor and increase in the currents elicited by low GABA concentrations.
Stiripentol	Third	Enhancement of inhibitory, γ-aminobutyric acid (GABA)ergic neurotransmission.
Cenobamate	Third	Modulation of the GABA_A_ channels, sodium currents, inhibition.
Fenfluramine	Third	Causes the release of serotonin by disrupting vesicular storage of the neurotransmitter, and reversing serotonin transporter function.

**Table 2:** Summary of studies on SNPs affecting AED transporters.

Study	Number of Participants	Drug	Gene	RefSNP	Clinical Effect
Shen et al., 2017	116	Oxcarbazepine	ABCB1	rs1045642	Higher concentration in the ABCB1 3435C > T CC carriers
Ma et al., 2015	184	Oxcarbazepine	ABCC2	rs2273697	Higher doses in the ABCC2 1249G > A carriers
Yao et al., 2022	125	Oxcarbazepine	ABCB1	rs1045642	Higher MHD CDR in the ABCB1 3435C > T CC carriers
Zhao et al., 2022	231	Lacosamide	ABCC2	rs2273697 rs717620	Lower lacosamide CDR in the ABCC2 1249G > A GA and AA genotypes and the ABCC2 24C > T CT and TT genotypes
Zhao et al., 2022	131	Lacosamide	ABCB1	rs2032582 rs1045642	Higher frequency of drug-resistance in the ABCB1 G2677T/A GT genotype. Higher CDR in the G2677T/A GG and ABCB1 C3435T CC genotypes. Higher plasma levels in the ABCB1 C3435T CC carriers
Zhou et al., 2015	140	Lamotrigine	ABCG2	rs114020 rs2231142 rs2622628	Lower CDR in the ABCG2 T > C TT carriers. Higher concentrations in the ABCG2 C > A CA and AA carriers
Shen et al., 2016	112	Lamotrigine	ABCG2 OCT1	rs2231142 rs628031	Higher lamotrigine levels in the OCT1 rs628031 AA and GG and in the ABCG2 rs2231142 AA carriers
Lovrić et al., 2012	222	Lamotrigine	ABCB1	rs1045642	Lower lamotrigine levels and DCR in 1236 CT and TT carriers
Stasiołek et al., 2016	271	Topiramate Oxcarbazepine Gabapentin Lamotrigine Levetiracetam	ABCB1	rs1045642	Higher incidence of drug resistance in the ABCB1 C3435T CC carriers
Ufer et al., 2011	208	Oxcarbazepine	ABCC2	rs2273697	Higher frequency of responders in the ABCC2 1249G > A carriers

**Table 3:** Summary of studies on SNPs affecting AED brain-targets.

Study	Number of Participants	Drug	Gene	Ref SNP	Clinical Effect
In et al., 2021	214	Sodium channel blockers	SCN1B	rs55742440	Higher frequency of allele C of SCN1B rs55742440 in the no-responders
Angelopoulou et al., 2017	200	Sodium channel blockers	SCN1A	rs3812718	SCN1A IVS5-91G > A GG carriers needed lower doses
Ma et al., 2015	184	Oxcarbazepine	SCN1A	rs3812718	SCN1A IVS5-91G > A variant allele carriers needed higher doses

**Table 4:** SNPs affecting the AED metabolizing enzymes.

Study	Number of Patients	Drug	Gene	Ref SNP	Clinical Effect
Lu et al., 2017	124	Oxcarbazepine	UGT1A9	rs2741049	Significantly lower MHD and worse seizure control in the UGT1A9 I399 C > T carriers.
Shen et al., 2017	122	Oxcarbazepine	UGT2B7	rs7439366	Higher doses in the UGT2B7 802T > C CC carriers.
Ma et al., 2015	184	Oxcarbazepine	UGT2B7	rs7439366	Higher doses in the UGT2B7 802T > C variant alleles carriers.
Petrenaite et al., 2022	317	Lamotrigine	UGT1A4 UGT2B7 UGT2B15	rs6755571 rs7439366 rs1902023	Lower lamotrigine ratios in the wild-type UGT1A4 70C > A C, UGT2B7 802T > C TT and UGT2B15 253G > T TT. 1.3-fold higher lamotrigine ratio in patients devoid of UGT2B17 gene.
Chang et al., 2014	106	Lamotrigine	UGT1A4	rs2011425	Higher drug concentration and better therapeutic efficacy in the UGT1A4 142T > G TT.
Du et al., 2016	102	Lamotrigine	UGT1A4	rs2011425	Higher drug concentration and better therapeutic efficacy in UGT1A4 142T > G TT.
Ahn et al., 2022	111	Lacosamide	CYP2C9 CYP2C19	rs1057910 rs4244285 rs4986893	Higher drug levels and CDR and lowest proportion of lacosamide-resistant patients in the PM group.
Okada et al., 2008	99	Zonisamide	CYP2C19	rs4244285 rs4986893	Lower zonisamide clearance in the heterozygous extensive and poor metabolizers.

## Data Availability

Not applicable.
